# Could Coronavirus Disease 2019 (COVID-19) Render Natural Immunity to Re-infections? A Spotlight on the Therapeutic Pipeline

**DOI:** 10.3389/fimmu.2020.01294

**Published:** 2020-06-05

**Authors:** Muhammad Abbas Abid, Loren Nunley, Muhammad Bilal Abid

**Affiliations:** ^1^Department of Otolaryngology—Head & Neck Surgery, Johns Hopkins University School of Medicine, Baltimore, MD, United States; ^2^Division of Infectious Diseases, Department of Medicine, Medical College of Wisconsin, Milwaukee, WI, United States; ^3^Division of Hematology/Oncology, Department of Medicine, Medical College of Wisconsin, Milwaukee, WI, United States

**Keywords:** COVID-19, adaptive immunity, convalescent plasma, vaccine development, antibody-dependent enhancement (ADE)

The pandemic of Corona Virus Disease 2019 (COVID-19) caused by a novel coronavirus, severe acute respiratory syndrome coronavirus 2 (SARS-CoV-2), is continuing to cause substantial loss of life and economic damage globally. Epidemiological studies have indicated that majority cases are mild and self-limiting. However, the mortality rate ranges between 2 and 20% depending upon patient's age, demographic factors, and comorbidities (1–4)^1^. Thus, far, the largest study of 72,314 patients from China reported that 81% of cases were mild with a case fatality rate of 2.3%. The study further showed that a subgroup of 5% of cases had a more severe illness—respiratory failure, septic shock, coagulopathy, and multiorgan dysfunction—and among those mortality was nearly 50%.

The epidemiological studies of COVID-19 patients available thus far underscore the heterogeneity of clinical presentation as well as the unpredictable nature of its progression to cytokine storm and acute respiratory distress syndrome (ARDS)–terminal events that lead to mortality associated with COVID-19 (1–3). Most patients who succumb to COVID-19 develop severe illness and are reported to have other comorbidities, immunosenescence, or are immunosuppressed (3–7)^1^.

In a desperate attempt to curb mortality in severe COVID-19, several immune- and non-immune-based therapeutic strategies, both investigational and repurposed, are being utilized including convalescent plasma, anti-microbial, anti-inflammatory, and immunomodulatory agents (8–14). However, no evidence exists related to the safety and efficacy of these agents and current measures are akin to “shooting in the dark” with a hope that “something will work.” For instance, the most favored and commonly used drug worldwide in the initial phase of the outbreak is now shown to be non-efficacious and, potentially, more toxic. In an open-label, randomized controlled trial of 199 PCR-confirmed COVID-19 patients, HIV-1 protease inhibitor, lopinavir-ritonavir (that showed *in vitro* activity against SARS-CoV-1), did not demonstrate any impact on clinical improvement, mortality or viremia, in comparison to supportive medical management (9). The other repurposed drugs that were expected to change the course of illness have also not demonstrated a clear signal thus far. In the particular cases of hydroxychloroquine (HCQ) and remdesivir, no clear clinical benefit has been demonstrated in several studies reported thus far. Studies have also suffered from uninterpretable or flawed trial designs (heterogeneous comparator arms), small sample size, either having a clinically oriented outcome or not demonstrating clinical benefit, or did not have sufficient data to demonstrate safety (e.g., baseline and serial electrocardiograms in the case of studies conducted to evaluate HCQ). In an open-label, non-randomized trial involving 26 patients receiving hydroxychloroquine (HCQ) (a fraction of patients also received azithromycin) and 16 unmatched controls, HCQ did not demonstrate any changes in patients' outcomes, despite increased viral clearance (11). Similarly, remdesivir has been another much-anticipated antiviral agent that still needs to demonstrate efficacy through a well-designed, randomized controlled trial. Clinical data from a non-randomized, single arm study, conducted via a compassionate use program, involving 53 patients with severe COVID-19 showed clinical improvement in 36 of these (68%) (14).

The kinetics and robustness of the immune response to COVID-19 are not known. However, given the critical need to understand the immune mechanisms of the rapidly crippling pandemic, evidence from other similar viruses, and prior coronaviral outbreaks (SARS-CoV-1 and MERS-CoV) may be extrapolated. For instance, McElroy et al. demonstrated a sustained and robust immune response to Ebola virus, comprising of B-cells, CD4+, and CD8+ T-cells. The group further identified viral proteins targeted by T-cells (15). Other groups also demonstrated the critical role of follicular helper T-cells (T_FH_ cells) and antibody-secreting cells (ASCs) toward development of immunity after infection as well as vaccination (15, 16). In a clinical study that demonstrated the kinetics of humoral immune response in 20 patients who developed severe acute respiratory syndrome (SARS), the SARS-CoV-1-specific IgG antibody was shown to last for a considerable duration during the convalescent phase (17). The IgG peaked at 1:640 during 12th week post-infection (with the cutoff value for the positive result being 1:10). Interestingly, Tang et al. showed in a clinical study involving SARS patients that SARS-CoV-1-specific IgG as well as memory B-cells may disappear, however, SARS-CoV-1-specific memory T cells could be detected as late as 6 years post-infection (18). Memory T-cells in murine studies have been shown to enhance the innate immune response in both SARS-CoV-1 and Middle East respiratory syndrome (MERS)-CoV demonstrating the potential of a vaccine that could exploit this cross-reactivity and may hold promise for efficacy across betacoronaviruses (19).

As of this writing, the only report that underpins adaptive immune response to COVID-19 is of a 47-years-old female with no past medical history, whose symptoms started 7 days after arriving in Australia from Wuhan, China. In the case report of a patient with mild COVID-19, Thevarajan et al. showed that a robust adaptive immune response ensued, comprising of effector T-cells (T_FH_ cells, activated CD4+ T-cells, and CD8+ T-cells), ASCs and SARS-CoV-2-binding antibodies (20). Interestingly, another study in rhesus macaques has suggested that primary SARS-CoV-2 may render natural immunity and could protect from subsequent infections (21).

In terms of humoral immune responses to SARS-CoV-2, preliminary data suggests that more than 90% of immunocompetent adults developed antibodies directed against SARS-CoV-2. However, the neutralizing capability, protection bandwidth, and longevity of response remain to be determined. In an inpatient cohort of 173 PCR-confirmed COVID-19 patients, Zhao et al. demonstrated that the seroconversion rate for total antibodies, IgM, and IgG was 93, 83, and 65%, respectively (22). Kissler et al. utilized mathematical estimates of seasonality and cross-immunity of two seasonal coronaviral strains, most closely related to SARS-CoV-2—betacoronaviruses HKU1 and OC43—and predicted that the immunity may last for a year (23). The group also projected that recurrent seasonal (winter) outbreaks may occur after the initial intense pandemic. The data from prior coronaviral outbreaks suggest that the immunity may last for several years, however, there is currently no evidence on cross-immunity between distinctive coronaviral strains (18). Longitudinal studies analyzing the robustness and longevity of the immune response to SARS-CoV-2 are desperately needed. As the pandemic intensifies and herd immunity develops, it is imperative to concurrently expand critical care infrastructure, reinforce mitigation, and containment strategies, advance vaccine development initiatives and further the therapeutic pipeline.

A detailed understanding of this emerging data related to COVID-19, in the context of prior robust evidence for other viruses, is critical, particularly when convalescent plasma therapy is increasingly being used to urgently counter the COVID-19-associated mortality and urgent vaccine development is imperative. The single report that underpins the development of a robust immune response, akin to those developed in other similar viral infections, is from a patient who was young with no comorbidities and developed a mild illness (20). Most patients who die of COVID-19 develop severe illness and are reported to have other comorbidities or are immunosuppressed (1, 3, 5–7). Immune-based treatments, such as convalescent plasma therapy, may be strategically utilized once data evaluating potential risk factors that lead to immune paresis in severe COVID-19 becomes available. Furthermore, empiric usage of convalescent plasma may even be detrimental in select patients as “antibody-dependent enhancement (ADE)” may lead to a more severe subsequent infection. ADE may occur if a patient has pre-existing antibodies to a virus that cross-react, do not neutralize, and enhance infection against another virus, or another serotype of the same virus. The phenomenon is best described in patients with pre-existing immunity to Dengue virus and may enhance Zika virus infection and lead to increased disease severity (24, 25). No data exists thus far suggesting or refuting a similar immunological counter-reactivity in COVID-19. However, *in vitro* evaluation of the mechanism of ADE in SARS-CoV-1 revealed that macrophages and monocytes are the culprit immune cells via their Fc receptors (FcR) (26). Hence, these FcR-bearing cells might facilitate viral entry via Fc domains of antibodies and their non-neutralizing nature could mount a pro-inflammatory response and lead to immune dysregulation (27). Although Dengue and Zika viruses are more closely related to each other, with substantial antigen overlap, in contrast to coronavirus group of viruses which is restricted to bats (SARS-CoV-1) or perhaps to a geographical location (MERS-CoV), caution still needs to be exercised given that coronavirus has crossed the xenographic barrier thrice in the last two decades and caused substantial mortality in humans. The second study in rhesus macaques highlighting immune responses to COVID-19 is limited by the sample size. The study involved four rhesus macaques of which only one was followed after re-infection and did not develop viremia or severe illness (21).

As the early evidence emerges, several vaccines are being developed with variable targets. Although vaccine development and a robust therapeutic pipeline are of critical importance currently, it is equally important that the emerging data is critically analyzed, and the sense of urgency does not avert clinicians from their Hippocratic Oath of “first do no harm.” The race against COVID-19 must not extract but the best out of us!

## Author Contributions

MBA conceived of the idea, performed the literature search, and wrote the manuscript. LN and MAA performed the literature search and co-wrote the manuscript. All authors approved the final version of the manuscript AND agree to be accountable for all aspects of the work in ensuring that questions related to the accuracy or integrity of any part of the work are appropriately investigated and resolved.

## Conflict of Interest

The authors declare that the research was conducted in the absence of any commercial or financial relationships that could be construed as a potential conflict of interest.

## Footnotes

^1^Available online at: https://www.cdc.gov/coronavirus/2019-ncov/need-extra-precautions/groups-at-higher-risk.html (accessed April 1, 2020).
